# Comparison of Motor Activity and Sleep in Patients with Complex Partial Seizures on Levetiracetam Treatment and a Group of Healthy Subjects

**DOI:** 10.1155/2007/832769

**Published:** 2007-08-22

**Authors:** Hikmet Yilmaz

**Affiliations:** Section of Epilepsy and Sleep DisordersDepartment of NeurologySchool of MedicineCelal Bayar UniversityManisaTurkey

**Keywords:** Actimeter, levetiracetam, nap episodes, total activity score, drowsiness, sleep efficiency

## Abstract

*Purpose:* Levetiracetam-treated patients commonly report daytime drowsiness, fatique, asthenia and decreasing of motor activity. However the origin of these reported side effects are still debated, we aimed to clarify effect of levetiracetam on sleep. Therefore this prospective study was conducted to evaluate the effects of levetiracetam on motor activity, amount and continuity of sleep and napping.

*Methods:* Various tests were performed on twenty two patients treated with levetiracetam (10 monotherapy, 12 add-on therapy) at least three days before the initiation of treatment, and consecutively for five to eight days at the third week of treatment. These tests included sleep logs, Pittsburgh Sleep Quality Index, Epworth Sleepiness Scale, Modified Maintenance of Wakefulness Test and actimetric measurements. In order to evaluate the sleep behavior of these patients the following sleep parameters were estimated: bedtime, wake-up time, sleep-onset time, sleep-offset time, sleep latency, total sleep time, wake time after sleep onset, fragmentation index, total activity score, nap episodes, total nap duration and sleep efficiency. Twenty members of staff from our hospital (Doctor, nurse, secretary, civil servant etc.) were evaluated as control subjects in the study.

*Results:* After three-week treatment with levetiracetam (in particular with add-on therapy), Epworth Sleepiness Scale scores, napping episodes and total nap durations increased and sleep latencies decreased. While durations of Modified Maintenance of Wakefulness Test and total activity scores decreased. However the total sleep time and the sleep efficiency did not show any difference from the pre-treatment values.

*Conclusions:* Our results suggest that levetiracetam leads to drowsiness by decreasing the daily motor activity and increasing the naps; however this agent does not have any major effects on total sleep time and sleep efficiency during night. Actimetric analyses give information about continuity of sleep and sleep/wake states however does not give satisfactory information about architecture of sleep. In order to determine the effects of levetiracetam on the sleep architecture we need similiar protocol studies by full night polysomnography.

## 1. Introduction

LEV-treated patients with epilepsy commonly complain of sleep disruption and report daytime sleepiness, somnolence, fatigue and asthenia [1,2,6,17,20]. In mono- and polytherapy trials, LEV has been reported

ISSN 0953-4180/07/$17.00 © 2007 – IOS Press and the authors. All rights reserved to cause somnolence in 4–15% of the patients, leading to drug discontinuation in 10% of patients [2]. Bell et al. [2] evaluated the effects of LEV (1,000 mg in a single dose) on sleep in healthy volunteers and epileptic patients on carbamazepine monotherapy. In this study by Bell et al., LEV was observed to produce an increase in sleep both in volunteers and epileptic patients. Moreover, LEV increased REM sleep latency (in volunteers) and decreased the time spent in slow wave sleep (SWS) (in patients). Volunteers and patients reported that sleep was of a better quality; both groups felt less alert on awakening in the morning, but objective daytime sleepiness was not assessed. Recently, Khatami et al. [9] reported a patient with brain tumor and epilepsy in whom increased sleep needs developed during an add-on treatment with LEV.

The relationship between the sleep efficiency, motor activity, naps and LEV has not been thoroughly investigated. This prospective study was conducted to evaluate the effects of LEV on motor activity, naps and sleep efficiency. This is the first study to use actigraphic nap analytical methods comparing LEV-treated patients with a control group.

## 2. Materials and methods

Thirty-three LEV-treated patients with complex partial seizures and twenty healthy subjects participated in this study. Subjects were in self-reported good physical health, with no sleep disorder, well controlled with no seizures and during this study were not taking psychotropic medications or any other medications known to interfere with normal sleep. Patients with minor health problems such as mild hypertension or mild arthritis were not excluded from the study. A brief psychiatric screening (Hamilton Depression Rating Scale) [8] was performed on each subject. A score of less than 7 was required for inclusion in the study. The study was approved by the Institutional Ethic Committee. All participants gave their written informed consent. All subjects were instructed to complete a standard sleep diary. Sleep assessment questionnaires were completed by the patients and control subjects in the morning hours (from am 8 to am 11 h). Pittsburgh Sleep Quality Index (PSQI), Epworth Sleepness Scale (ESS), Modified Maintenance of Wakefulness Test (MMWT) and actimetric measurments were used for sleep evaluation.

PSQI is a subjective measurement that evaluates the sleep quality and disturbances during the last month. All subjects answered 19 questions and results were assessed as "good sleep quality" for scores $\leqslant 5$ and as "bad" for scores $>5$ [19]. Subjects with a score $>5$ were excluded from the study. Also, patients have seizures during the study were excluded. After all these exclusions, the study was continued with twenty two LEV-treated patients and twenty healthy members of staff from our hospital (Doctor, nurse, secretary, civil servant etc.). Ten of these 22 patients were using another drug (carbamezepine, oxcarbazepine, valproate and diphenil hidantoin) before LEV treatment.

ESS was used to evaluate the overall level of self-reported daytime sleepiness over the last week. The scores (24 in total), which were equal to or grater than 10 were assessed as sleepiness [10]. ESS was performed prior to LEV treatment and repeated at the third week of LEV treatment (2000 mg/day) for the study group. This test was performed once in control subjects.

The MMWT is a laboratory-based daytime polysomnographic test that measures the ability of the patient to stay awake in a darkened, quiet environment during the daytime. It is used in adult subjects for the assessment of effects of drugs on sleepiness [11]. Sleep logs, visual observations or actimeters may be used prior to MWT (instead of full-night PSG) [7,12]. MMWT was performed by forcing the subjects to maintain their wakeful state in a silent and dim room for a total of four 20-minute periods at 2-hour intervals. The subjects was then told to stay awake as long as possible, the test was initiated and the subjects were monitored by an EEG-technician. Monitored electroencaphalographic variables were revealed from C3, C4, O1 and O2; electrooculogram (EOG, right and left outer canthi) and electromyogram (EMG, submental) according to standardized procedures [15]. Sleep onset was defined by any single 30 seconds epoch of sleep stage 2, 3, or 4, or REM-sleep according to the criteria of Rechtschaffen and Kales [15]. The trial was terminated after 20 minutes if no sleep had occured. Sleep latency was determined from lights off time (dimmed lights and door closed) to the first epoch of sleep on the EEG. Recordings were done using an Embla Recording Systems with *somnologica* software (Medcare, Reykjavik, Iceland). Average wakefulness time was calculated by dividing the total wakeful time that cases were able to maintain for the four periods [18]. Thus drowsiness of the subjects was evaluated. MMWT was performed prior to LEV treatment and repeated at the third week of LEV treatment (2000 mg/day) for the study group. This test performed four times a day in the control subjects.

### 2.1. Actimetric procedures

The actimeter is designed for long-term monitoring of gross motor activity in humans. It has an accelerometer capable of sensing motion with a minimal resultant force of 0.01 g [16]. Participants wore a wrist actimeter (Actiwatch, AW64®, Mini-Mitter Co. Inc., Bend, OR, USA) on their nondominant wrist for the examination of their motor activity and sleep analysis. Actimetric analysis was performed for a minimum of 3-day period prior to LEV treatment. This analysis was repeated during a five to eight-day period at the third week of LEV treatment (2000 mg/day) for the study group. Control group subjects continuously wore the wrist actimeters for a seven-day period. Written and oral instructions for using the actimeter were provided at each measurement period. Subjects were told to press the event marker button to indicate the timing of an event (eg, bedtime, wake time) or to demarcate an interval of time (eg, when the exercise begins and ends, when a medication is taken, etc). They kept daily sleep logs during the period of actigraphy. These sleep logs were used to record daily bed times, times of sleep onset, frequency and duration of awakenings during the sleep period, final awakening time, and periods of inactivity during the day in order to obtain data on sleep latency, frequency of prolonged awakenings, and total sleep time. These data were used to confirm and support the interpretation of the event marker data obtained with the wrist actimeters. All actimeters were calibrated prior to use and set to collect data at 30-seconds periods with a maximum frequency of 32 Hz according to the criteria used by Sadeh [16]. Device sensitivities were determined in order that a motor activity $\geqslant$5 per sec. indicating the wakefulness of the subject. Actimeter data were analysed by computer. A minimum of 10 min without motor activity after pressing the event marker was determined as "sleep onset". Counts of motor activities less than 5 per sec. were assessed as sleep period in the analysis of napping. Minimum nap time was determined to be 15 minutes as reported by Brooks et al. [5]. The primary sleep period was identified based on the times that participitans pressed the event button on the actimeter, in the evening at lights out and in the morning after waking up, making it possible to log these events. For evaluating nocturnal sleep, the following sleep parameters were estimated: bedtime, wake-up time, sleep-onset time, sleep-offset time, sleep latency (SL), total sleep time (TST), wake time after sleep onset (WASO), and sleep efficiency (SE). Bedtime and wake-up time were determined from the activity scores and sleep logs. Nocturnal sleep interval for scoring was set from bedtime to wake-up time. SE [(nocturnal sleep time/time in bed) $\times$ 100] and the other sleep parameters were obtained from the actigraphic sleep estimates. Sleep onset and offset was defined as the first and last sleep epoch within the nocturnal sleep interval, respectively. Naps were analyzed in reference to each day's wake-up time and the subsequent bedtime.

### 2.2. Statistical analysis

We used SPSS 10.0 for Windows for statistical analyses. All the sleep parameters were compared between control group – pretreatment group and pre-treatment group – post-treatment group by Mann-Whitney U test. Significance was defined at $p < 0.05$.

## 3. Results

The mean age of the study group and control group was 38.8 (range: 18–72) and 40.0 (range: 20–71), respectively. There was no statistically significant difference in age or sex between the control and study groups ($p > 0.05$). Comparisons of the sleep parameters between the groups are presented in table. The control group and the pre-treatment group didn't show any difference in sleep parameters. After 3 weeks of LEV therapy (in particular at add-on therapy group); MMWT durations were shorter than the pre-treatment group values ($p < 0.001$).

### 3.1. Motor activity

Actigrams were used to evaluate the motor activities of the subjects. Subjective reports of activity via activity diaries and sleep logs assisted in understanding and interpreting the actigrams. After completing the actimetric analysis for all the subjects, actigram data were compared between the groups. The decrease in total activity observed in post-treatment group was statistically significant (in monotherapy group, $p < 0.05$; in add-on therapy group, $p < 0.001$) and noticeable. Activity counts are shown at the right side of the diagram (Fig. 1).

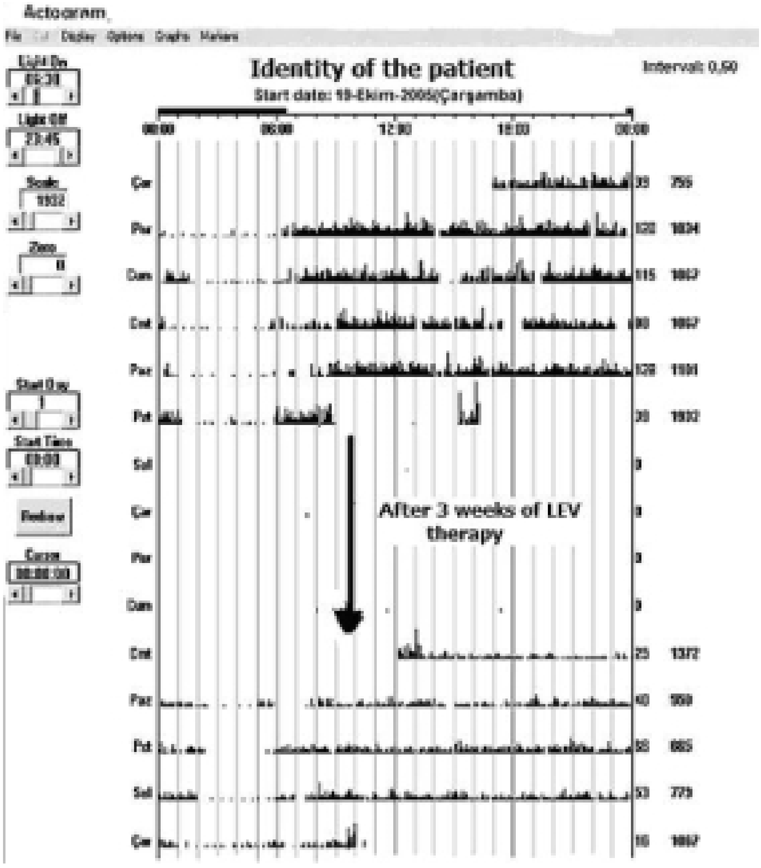

Fig. 1. A sample actogram. First period consists of 6 consecutive days pre-treatment and the second period consists of 5 consecutive days after three weeks of LEV monotherapy. Decreasing of total activity observed in the second period is noticeable. Activity counts are shown at the right side of diagram.

### 3.2. *Nap episodes and duration*

Nap term is used to describe short periods of rests where activity decreased and reactions to external stimuli were reduced. Comparing the groups with respect to nap episodes and total nap duration, both the nap episodes and the total nap duration were observed to be significantly increased in post-treatment group (in monotherapy group, $p < 0.05$; in add-on therapy group, $p < 0.001$). Three subjects in the control group and three subjects in pre-treatment group had at least one nap which lasted more than 15 minutes (15% of control group and 13.6% of the pre-treatment group) while eighteen subjects in the post-treatment group (in monotherapy group, seven subjects; in add-on therapy group eleven subjects) showed at least one nap lasting more than 15 minutes (81.8%). Distribution of the nap episodes in post-treatment group was interesting (Fig. 2). We observed that, nap episodes were clustered in the morning (from 9 to 11 am) and evening period (from 9 to 11 pm).

### 3.3. *The effects of naps on nocturnal sleep*

While there was no significant difference between the control and pre-treatment groups in sleep parameters; we found that the SL in post-treatment group (in particular at add-on therapy group) was shorter compared to those of the control and pre-treatment groups (Table 1). The relative shortness of SL may be related to the evening naps of the post-treatment group. However the other sleep parameters such as TST and SE were not affected by naps.

## 4. Discussion

Levetiracetam (LEV; Keppra, UCB Pharma, Belgium), the S-enantiomer of alpha-ethyl-2-oxo-1-

Table 1
Sleep parameters of LEV treated patients and control group

| Parameters | Control group $n = 20$ | Before LEV treatment $n = 22$ | | After three weeks of LEV treatment $n = 22$ | |
|---|---|---|---|---|---|
| | | Using no antiepileptic $n = 12$ | Using an antiepileptic $n = 10$ | Monotherapy $n = 12$ | Add-on therapy $n = 10$ |
| ESS (point) | 2.45 ± 0.69 (0.00–6.00) | 2.48 ± 0.70 (0.00–6.00) | 2.53 ± 0.75 (0.00–6.00) | 2.60 ± 0.90 (0.00–7.00) | 2.62 ± 0.99 (0.00–7.00) |
| MMWT (min) | 18.54 ± 0.93 (14.00–20.00) | 18.88 ± 0.64 (14.00–20.00) | 18.94 ± 0.67 (14.00–20.00) | 17.45 ± 0.82 (14.00–19.00) | 14.10 ± 0.93* (14.00–19.00) |
| SL (min) | 16.13 ± 7.45 (9.00–30.00) | 15.50 ± 8.30 (10.00–30.00) | 15.58 ± 8.35 (10.00–30.00) | 15.00 ± 6.24 (8.00–30.00) | 14.89 ± 6.05 (8.00–30.00) |
| TST (hour/night) | 7.18 ± 0.43 (6.25–8.06) | 7.15 ± 0.62 (6.00–8.05) | 7.19 ± 0.66 (6.00–8.05) | 7.19 ± 0.98 (6.05–8.42) | 7.20 ± 0.88 (6.20–8.20) |
| Total activity score (number/night) | 9608 ± 7200 (1334–22280) | 9840 ± 7323 (1034–23028) | 9857 ± 7357 (1034–23028) | 7779 ± 7861** (1243–23591) | 5980 ± 8200* (3421–23571) |
| WASO (min) | 14.13 ± 2.02 (6.00–41.00) | 14.54 ± 2.00 (5.00–40.00) | 14.62 ± 2.02 (5.00–40.00) | 15.14 ± 2.71 (6.00–40.00) | 15.17 ± 2.53 (6.00–41.00) |
| Fragmentation Index (number/day) | 8.43 ± 1.23 (1.90–17.00) | 8.40 ± 1.20 (2.00–17.80) | 8.45 ± 1.22 (2.00–17.80) | 8.54 ± 2.30 (1.90–18.50) | 8.90 ± 2.01 (2.02–19.90) |
| Sleep Efficiency (%) | 96.65 ± 15.21 (79.00–99.00) | 96.64 ± 11.90 (79.00–98.00) | 96.75 ± 12.02 (79.00–98.00) | 95.20 ± 14.92 (78.40–98.20) | 95.00 ± 11.17 (78.00–97.00) |
| Daytime napping episodes (number/day) | 2.42 ± 0.29 (1–4) | 2.44 ± 0.42 (1–4) | 2.66 ± 0.46 (1–4) | 3.45 ± 1.80** (2–7) | 6.70 ± 1.20* (2–11) |
| Total nap duration (min/day) | 22.00 ± 4.13 (15.00–31.00) | 23.00 ± 3.90 (15.00–35.00) | 23.04 ± 4.04 (15.00–35.00) | 38.00 ± 8.23** (24.11–54.00) | 59.00 ± 9.32* (24.91–81.07) |

*$p < 0.001$, **$p < 0.05$ Mann-Whitney U test.

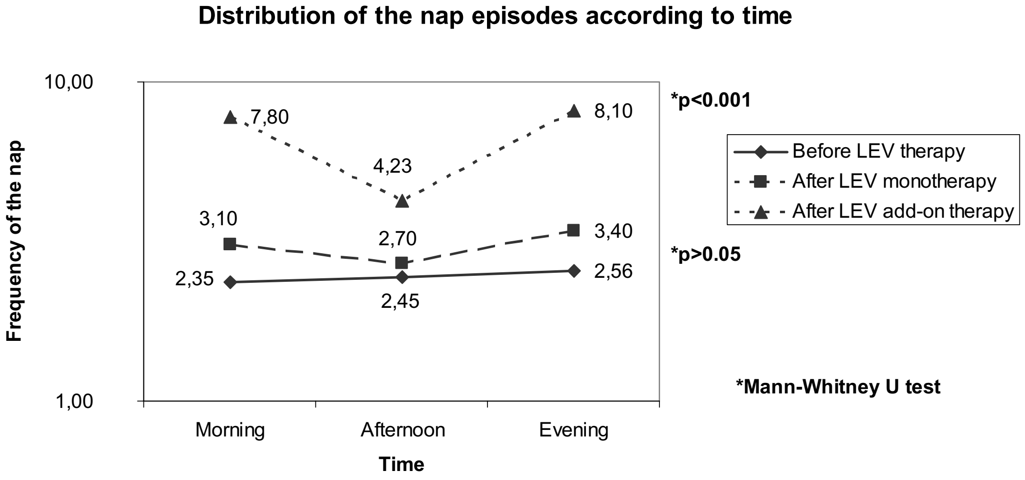

Fig. 2. Distribution of the nap episodes by time. Nap episodes generally appeared to be related to the LEV administration. Perhaps, owing to this characteristic of napping episodes, sleep latencies in the post-treatment group are shorter than those in the pre-treatment group.

pyrrolidine acetamide is an antiepileptic drug approved as adjunctive therapy in partial-onset seizures with or without secondary generalization in epilepsy [3,4, 13,21]. LEV has been reported to cause sleepiness, somnolence, fatigue, asthenia and decreasing of motor learning, thereby leading to drug discontinuation in some of the patients [1,2,6,17,20]. However, objective data on the nature and/or severity of these findings observed with LEV are missing in the literature. Thus this study was designed to examine the effects of LEV on motor activity, naps and sleep efficiency. Our study showed that LEV, therapeutic doses for treatment of epilepsy, caused an increase in nap episodes and nap durations, and a decrease in SL and motor activity. Unchanged TST and SE were also considered as noteworthy findings. Decrease of MMWT durations also supported that LEV caused a decrease in motor activity and an increase in nap episodes and nap durations in the post-treatment group (in particular add-on therapy group). These results are partially consistent with the previous findings from the studies of Khatami et al. [9], which reported LEV-associated hypersomnia with a decrease observed in daytime SL.

More than 80% of the patients in the post-treatment group were reported to have a new or previous diagnosis of naps. Distributions of these naps was interesting with the naps clustering in morning and evening periods (Fig. 2). We observed that these nap periods were related to the times of LEV administration (patients had received LEV two times, at 8 am and 8 pm) of the patients. LEV exerts its antiepileptic effect by suppressing excitatory system and promoting the GABAergic system [3,4,13,17,21]. LEV is rapidly and almost completely absorbed following oral administration with peak plasma concentrations occurring in about 1 hour [14]. Based on this data, we may conclude that, morning doses of LEV caused an increase in the morning naps and a decrease in the motor activity while evening doses of LEV led to an increase in the evening naps and a decrease in motor activity and SL.

We do not have sufficient data for the effects of LEV on sleep yet. Actimetric analyses give information about continuity of sleep and sleep/wake states however does not give satisfactory information about architecture of sleep. Our results suggest that LEV therapy (in particular at add-on therapy) decreases daytime motor activity, increases naps and decreases SL while it does not have any major effects on TST and SE. However TST did not seem to be affected, we need similiar protocol studies supported by full night polysomnography to determine changes in night time sleep architecture.

## Abbrevations

LEV: Levetiracetam
REM: Rapid eye movements
SWS: Slow wave sleep
PSQI: Pittsburgh Sleep Quality Index
ESS: Epworth Sleepness Scale
MMWT: Modified Maintenance of Wakefulness Test
TST: Total sleep time
WASO: Wake after sleep onset
TAS: Total activity score
SL: Sleep latency
SE: Sleep efficiency
FI: Fragmentation index
